# Characterization of Anti-bacterial Compounds from the Seed Coat of Chinese Windmill Palm Tree (*Trachycarpus fortunei*)

**DOI:** 10.3389/fmicb.2017.01894

**Published:** 2017-10-04

**Authors:** Shakeel Ahmed, Huimin Liu, Aqeel Ahmad, Waheed Akram, Eman K. N. Abdelrahman, Fengming Ran, Wuling Ou, Shuang Dong, Qian Cai, Qiyun Zhang, Xiaohua Li, Sheng Hu, Xuebo Hu

**Affiliations:** ^1^Laboratory of Drug Discovery and Molecular Engineering, Department of Medicinal Plants, College of Plant Science and Technology, Huazhong Agricultural University, Wuhan, China; ^2^National-Regional Joint Engineering Research Center in Hubei for Medicinal Plant Breeding and Cultivation, Huazhong Agricultural University, Wuhan, China; ^3^Medicinal Plant Engineering Research Center of Hubei Province, Huazhong Agricultural University, Wuhan, China; ^4^Hubei Cancer Hospital, Wuhan, China

**Keywords:** medicinal plant, anti-bacterium, 2, 4, 5-triacetoxybiphenyl, 1-(4-Fluorophenyl)-2-(methylthio)-1H-imidazole-5-carboxylic acid, *Trachycarpus fortunei*

## Abstract

The increasing of multidrug resistance in bacterial associated infections has impaired the current antimicrobial therapy and it forces the search for other alternatives. In this study, we aimed to find the *in vitro* antibacterial activity of seed coat of *Trachycarpus fortunei* against a panel of clinically important bacterial species. Ethanolic extracts of target tissues were fractionated through macro porous resin by column chromatography, using ethanol as an organic solvent with a concentration gradient of 0–100%, each along with 20% concentration increment. The minimum inhibitory (MIC) concentrations of all fractions were measured. It is found that 20% ethanolic fraction showed the most significant inhibition against tested bacterial species. All fractions were analyzed by Ultra-Performance Liquid Chromatography/mass spectrometry (UPLC/MS) and compounds were identified by comparing mass spectra with standard libraries. By pairing the identified compounds from different fractions with the antibacterial activity of each fraction, it was shown that compounds stearamide (7), 1-(4-Fluorophenyl)-2-(methylthio)-1H-imidazole-5-carboxylic acid (9) and 2,4,5 triacetoxybiphenyl (10) topped in the list for anti-bacterial activity. Further experiment with pure chemicals verified that compounds 9 and 10 have antibacterial activity against Gram-negative bacteria. Whereas, the lowest MIC value (39.06 μg/mL) was obtained by compound 10 against *Staphylococcus epidermidis*. Hence, the seed coat of *T. fortunei* with its antimicrobial spectrum could be a good candidate for further bactericidal research.

## Introduction

Plant oriented antimicrobial agents’ exhibit immense therapeutic potential. Traditional medicinal plants have paved a path to discover novel pharmaceutical agents that are able to overcome the resistant developed by microorganisms (Girish and Satish, 2008). Plants are a rich source of secondary metabolites like flavonoids, tannins, terpenoids and alkaloids, which were found responsible for antimicrobial activities of various plants (Cowan, 1999). Different plant extracts and phytochemicals have antimicrobial properties and they are significantly used in therapeutic treatments. For many years’ immense study has been conducted on these phytochemicals and plant extracts to evaluate their anti-bacterial properties.

*Trachycarpus fortunei* (Hook.) H. Wendl. is a well-known member of Arecaceae (Palmae) family with accessible palmate fronds. It is known as “Chinese windmill palm” or “Chusan palm” in China (Chapman and Wang, 2013). *T. fortunei* is one of the most useful and widely grown palms in China. Recently research on this palm showed that its seeds, leaves, stems, roots and flowers contain many chemical components (Yunfa, 2005;Chen et al., 2012). Author and cooperative scientists have exploited the hemostatic drug “Xuean” and plant wax. The windmill palm has great potentialities, polyphenols, flavonoids and steroidal saponins in its seeds and leaves, which might be beneficial to cure heart and vascular diseases and tumors (Yunfa, 2005).

Metabolic profiling by UPLC/MS is widely used for various plant-derived products (Pongsuwan et al., 2008; Xie et al., 2008). UPLC/MS gives ultrafast separation and identification as a result of rapid, high-throughput and sensitivity (Kim et al., 2011). The ability to generate maximum results in less time by UPLC/MS has facilitated the real-time analysis of the complex samples with diverse chemical properties (Shen et al., 2005). However, purification of a specific chemical from a plant normally takes a long time and great efforts. Previously, pairing the richness of compounds from LC/MS and variations of different samples had helped identification of active components directly from a large data set (Keller et al., 2013). This strategy inspired us to combine the chemical information of the UPLC/MS from different fractions based on eluent polarity with their bioactivity. In this regard, we firstly evaluated the anti-bacterial potential of fractions of *T. fortunei* eluted by different concentrations of ethanol. Then, the compounds from each fraction were identified by UPLC/MS. A short list of potential chemicals that were responsible for the antibiotics was generated. Then a few compounds from *T. fortunei* that showed to be antibiotic were analyzed. To our knowledge this is the first study of anti-bacterial activity of chemicals from the seed coat of *T. fortunei*.

## Materials and Methods

### Plant Collection

The seeds of *T. fortunei* were collected from the campus of Huazhong Agricultural University (Wuhan, China) and were dried and stored at room temperature until further experiment. The seeds were taken from palm trees alone two sides of Xueyuan Road, beginning from the West entrance of the university to the crossing of Zhuyuan Road. The plants were identified by Prof. Xuebo Hu (Huazhong Agricultural University) to be *T. fortunei.*

### Extraction of *T. fortunei*

The plant material was extracted as described by Chatterjee et al. (2011) with some modifications. The seed coat of *T. fortunei* (2 kg) was exhaustively extracted three times with ethanol/water solution (70:30, v/v) for 2 h at 80°C at a revolution of 80 per minute under reflux. After 2 h, the solution was allowed to be cooled for 4 h with continuous agitation. Then the extract was removed from the apparatus, followed by paper filtration. After extraction, the ethanol in the solution was removed by rotary evaporator at 50°C and then sought to vacuum freeze drying. The extracts were weighed and then stored in a clean, dry and air-tight container with proper labeling in refrigerator at 4°C until further use.

### Fractioning of Seed Coat Extract of *T. fortunei*

Previously prepared extracts were further fractionated by column chromatography using ethanol and water solvent system Mbukwa et al. (2007). The extract was loaded on a glass column (10 cm × 250 cm) packed with the D101 macroporous resin (Sangon Biotech, China), and eluted with 0, 20, 40, 60, 80, and 100% ethanol (5 L each), to get different fractions of extract, respectively. Finally, each eluent fractions was evaporated under vacuum to obtain according fractions.

### Determination of Antibacterial Activity

Different fractions of seed coat extract of *T. fortunei* were screened against a collection of Gram-positive and Gram-negative bacterial strains, including *Staphylococcus aureus, S. epidermidis, Bacillus cereus, B. subtilis, Escherichia coli, and Pseudomonas aeruginosa*, which were obtained from the China General Microbiological Culture collection center (Beijing).

Antibacterial activity was evaluated by two different methods. At first, extracts activity was determined by measuring the inhibition zone against selected bacteria. Once antibacterial activity was confirmed, the minimum inhibitory concentration (MIC) was checked. MIC of different fractions of extract of *T. fortunei* plant were evaluated on the selected bacteria using rapid *p*-iodonitrotetrazolium chloride (INT) colorimetric assay according to method described by Kuete et al. (2008). Briefly, we dissolved different plant fractions in dimethyl sulfoxide (DMSO) methyl-4-hydroxybenzoate (MHB) with a ratio of (10:90). In a 96-well we added 100 μL of this solution to each well. Then we serially diluted two-fold for two times, followed by addition of 100 μL of bacterial inoculum that we already prepared in Mueller Hinton broth. Afterward, sealed plates were placed in an incubator for a period of 20–22 h at 37°C. The final concentration of inoculum was 1.5 × 10^6^ colony-forming unit /mL and it was less than 2.5% for DMSO in each well. Wells with inoculum and DMSO (2.5%) only were declared as negative control; whereas, streptomycin and tobramycin were used as positive control. After 20 h, we added 40 μL of INT (0.2 mg/mL) to each well and re-incubated for a period of 30 min. MIC is defined as the least concentration of extract that can inhibit the growth of bacterial species.

For the determination of MMCs, a portion of liquid (5 μL) from each well that showed no growth of microorganism was placed on MHA or NA and incubated at 37°C for 24 h. The lowest concentrations that yielded no growth after this sub-culturing were taken as the MMCs (Nyaa et al., 2009).

### Disk Diffusion Assay

Antimicrobial activity was explored by disk diffusion method as proposed by Bauer et al. (1966). Mueller Hinton Agar (MHA) and Nutrient Agar (NA) were used for the screening of *in vitro* antimicrobial activity. The MHA and NA plates were prepared by pouring 15 ml of molten media into sterile petri plates. These plates were allowed to solidify for 5 min, then swabbed uniformly with 0.1% inoculum suspension, and allowed to dry for 5 min. The 6 mm sterile disks were loaded with different concentrations (2.5 and 5 mg per disk) of extracts. Then these disks were placed on the surface of prepared medium and the compound was allowed to diffuse for 5 min and the plates were incubated for 22 h at 37°C. Then inhibition zones were measured with transparent ruler in millimeter around the disks. Each sample was analyzed in triplicate and values were presented as mm.

### UPLC/MS Analysis of Fractions

Organic fraction exhibiting the most antimicrobial behavior (20% ethanol fraction) was selected for identification of metabolites by performing UPLC/MS analysis. Purified fractions were filtered with filter papers of 0.45 μm pore size. Each of 50 μg of the sample was dissolved in 1 ml methanol (HPLC grade). The sample was then filtered through 0.2 μm filter paper (GH polypropylene 0.2 μm pore size, 47 mm diameter filter paper). Sample reaction solutions were transferred to UPLC vials (LC-MS certified 12 × 32 mm clear presilt combo, Waters) suitable for the UPLC-MS autosampler. A definite amount (30 μL) of filtered material was taken in a vial. Further, the samples were immediately analyzed by performing UPLC/MS analysis. Sample was prepared as described by Matsuura et al. (2008). Prepared samples were stored at 4°C before undergoing UPLC-MS operation. This precautionary measure was adopted to protect material from degradation.

The mass spectrometer was tuned according to the manufacturer’s manuals for optimal parameters for ion lenses, detector voltage and other settings. Sample reaction solutions were transferred to glass vials suitable for the UPLC/MS auto sampler. Each sample tube was closed immediately with crimps that contain a Teflon rubber seal. After 2 h, samples were injected into the UPLC/MS. The UPLC/MS analysis was carried out in a Waters’ (UPLC) coupled with Waters Q-ToF Premier Mass Spectrometer. UPLC conditions were used as described by (Matsuura et al., 2008). The LC column used was ACQUITY UPLC BEH C18 RF 1.7 μm, 2.1 × 50 column. Data analysis was carried out using MassLynx (Waters, Milford, MA, United States). Compounds were detected by comparing MS patterns with standard NIST Library. Metabolite peaks were quantified by area of targeted ion traces, and result of peak tables of all chromatograms were exported to MS Excel 14.0.

Obtained data were analyzed by performing both univariate statistics (*t*-test in Excel, ANOVA in MatLab) and multivariate statistics. The results of multivariate statistics were processed using minitab 17.1 (LEAD Tachnologies, Charlotte, NC, United States). Each experiment was repeated three times.

## Results

### Determination of Antibacterial Activity of Fractions Based on Eluent Polarity

In an effort to explore antibiotic substances, we have screened up to 200 kinds of plant extracts and found *T. fortunei* was one of the strongest one (data will be published separately). In this study, we aimed to screen the active compounds from *T. fortunei*. After extraction of the palm seed coat by ethanol/water solution (70:30, v/v), chemicals in the solution were dried out and then subjected to macroporous resin column separation by elution with 0, 20, 40, 60, 80, and 100% ethanol. The increasing ethanol concentration is adopted to differentiate the chemicals by polarity. Fractions were dried up by vacuum evaporation. It was found that fractions eluted by 80 and 100% ethanol yielded little chemicals, therefore chemicals eleuted by of 0, 20, 40, and 60% ethanol were used for further study.

**FIGURE 1:**
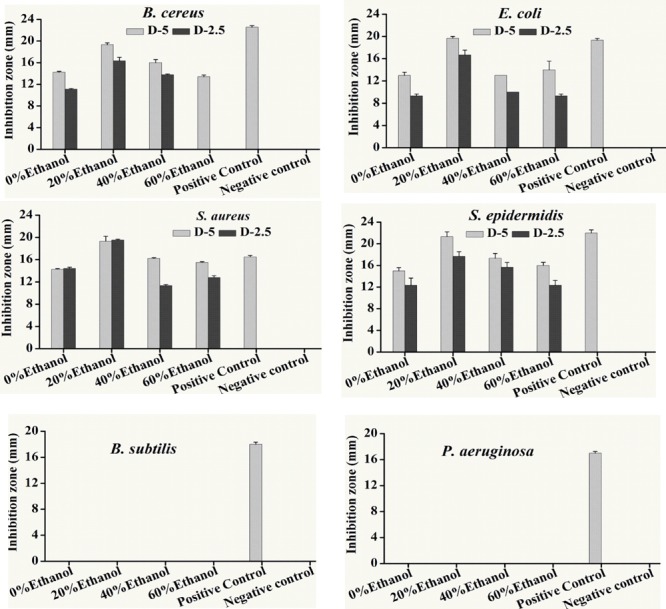
Antibacterial activity of different ethanolic fractions of seed coat materials of *Trachycarpus fortunei* against different pathogenic bacteria, i.e., *Staphylococcus aureus, Escherichia coli, Staphylococcus epidermidis, Bacillus cereus, Bacillus subtilis* and *Pseudomonas aeruginosa.* Extracted material was used in quantities of 5 and 2.5 mg. Antibacterial activity was assessed by analyzing inhibition Zones (mm). Bacterial species were incubated for 22 h at 37°C after the placement of disks soaked in different fractions. Values provided here are mean of three replicates. Statistical analyses were performed by using Statistix8.1 software and comparison among each treatment was based on one way ANOVA according to Tukey HSD test at *P* < 0.05 level significance.

Disk diffusion method was firstly used for determination of antibacterial activity of previously prepared fractions against various pathogens, i.e., *S. aureus, S. epidermidis, E. coli, P. aeruginosa, B. subtilis* and *B. cereus*. Resulted antibacterial inhibitory zones of all the fractions against different bacterial strains were listed in **Figure 1**. It can be seen that all fractions had antibiotic activity against *B. cereus, E. coli, S. aureus, S. epidermidis* and but not *P. aeruginosa* and *B. subtilis.* Furthermore, 20% ethanol fraction showed the highest anti-bacterial activity against most of the bacterial strains used in this study.

Minimum inhibitory was performed through a series of double dilution beginning from 1.25 mg/ml. *E. coli* growth was inhibited by almost all the ethanol and aqueous fractions of sample plant material but it showed the maximum inhibition from 20% ethanol fraction where the MIC = 156.25 μg/mL. All the fractions inhibited the growth of *S. epidermidis* with the same potency (MIC = 312.25 μg/mL). Inhibitory activity of *S. aureus* was the same as *E. coli*. Likewise, 20% ethanol also revealed the lowest value of MIC against *B. cereus* (312.55 μg/ml) among all the tested fractions (**Table 1**).

**Table 1:** Minimum inhibitory concentration (MIC, μg/mL) of all ethanolic fractions of seed coat material of *T. fortunei* against different bacteria.

Fractions	*S. aureus*	*E. coli*	*S. epidermidis*	*B. cereus*
0% ethanol	625	625	312.5	625
20% ethanol	156.25	156.25	312.5	312.5
40% ethanol	312.5	312.5	312.5	625
60% ethanol	312.5	312.5	312.5	625

Data in **Table 2** represented the minimum microbicidal concentration (MMC) values of the different fractions of seed coat extract of *T. fortunei* against reference bacterial strains. Although results of MIC and MMC showed variations among the tested bacterial strains but both the values were equivalent to each other in most of the cases, showing anti-bactericidal effect. Of all fractions of sample plant, the lowest MMC value (312.5 μg/mL) was recorded by 20% ethanol fraction against *S. aureus* and *S. epidermidis*.

**Table 2:** Minimum microbicidal concentration (MMC, μg/mL) of all ethanolic fractions of seed coat material of *T. fortunei* against different bacteria.

Fractions	*S. aureus*	*E. coli*	*S. epidermidis*	*B. cereus*
0% ethanol	1250	625	625	625
20% ethanol	312.5	625	312.5	625
40% ethanol	625	625	625	625
60% ethanol	625	625	312.5	625

### UPLC/MS Analysis of Fractions of *T. fortunei*

In order to search for the responsible chemicals for the antibiotic activity, we conducted UPLC/MS for all extractions. The Compounds that were identified from different ethanol aqueous fractions are compound 1–29. All the compounds were identified using NIST compound library on the basis of their mass spectra and their names are shown in **Table 3**.

**Table 3:** List of compounds identified by performing UPLC/MS analysis present in different ethanolic fractions of seed coat extract of *T. fortunei*.

Chemicals	Compound name	Peak area under fractions			
		0% ethanol	20% ethanol	40% ethanol	60% ethanol
1	Styrene	2125417.8			
2	Trimeprazine	11100000			
3	Evocarpine	4601170.9			
4	Magnoshinin	3342610.3			
5	Voacamine	2224015			
6	Protoveratrine A	5876061.4			
7	Stearamide		2.34E+08		
8	2,3-diphenyl- quinoxaline		2.73E+07		
9	1-(4-Fluorophenyl)-2-(methylthio)-1H-imidazole-5-carboxylic acid		7462685.7		
10	2,4,5-triactoxybiphenyl		3342082.3		
11	Phosphatidylethanolamine alkenyl		3236972		
12	Umbelliferone			34800000	
13	Podocarpic acid			6172905.9	
14	Buddledin A			15900000	
15	Pentamidine			5402284.9	
16	Istamycin C1			4696785.3	
17	Alpha-Oxo-benzeneacetic acid				2569008.7
18	Monodechloroaminopyrrolnitrin				7851935.1
19	Alpha-Amylcinnamaldehyde				6558895.8
20	Podocarpic acid				14100000
21	Stearidonic acid				22500000
22	Allogibberic acid				3336775.6
23	Abacavir				21700000
24	(9Z)-(13S)-12,13-Epoxyoctadeca-9,11-dienoic acid				12300000
25	(9Z,12Z)-(8R)-Hydroxyoctadeca-9,12-dienoic acid				5467640.6
26	Timolol				16800000
27	4,4-Difluoropregn-5-ene-3,20-dione				46100000
28	Dibenzo[h,rst]pentaphene				115000000
29	Aspidospermine				22700000

*Compounds were identified by comparing mass spectra with standard NIST library.*

**FIGURE 2:**
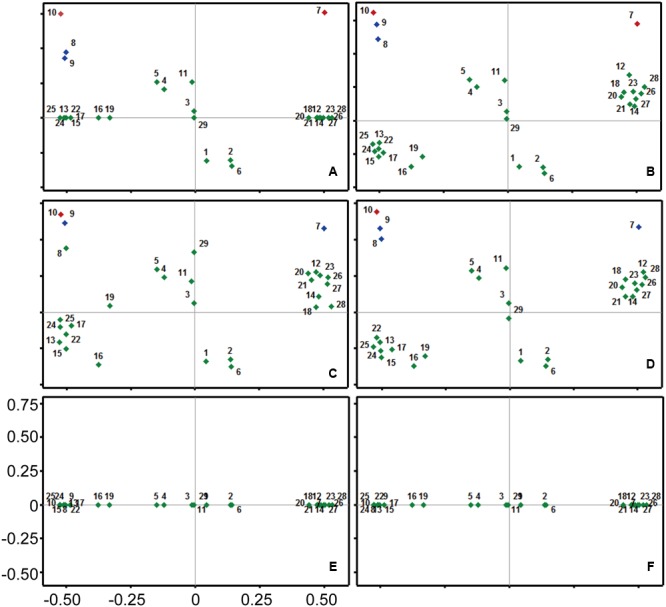
Principal component analysis (PCA) for all the chemicals of fractions of *Trachycarpus fortunei* against different pathogenic bacteria. **(A)** *Staphylococcus aureus*, **(B)** *Escherichia coli*, **(C)** *Staphylococcus epidermidis*, **(D)** *Bacillus cereus*, **(E)** *Bacillus subtilis*, **(F)** *Pseudomonas aeruginosa.* Coefficient for antibacterial activity has been mentioned on *Y*-axis, while scale on *X*-axis represents the affinity of detected compounds with ethanol concentration in extraction solvent. Compounds are numbered from 1 to 29. Details of compounds are presented in **Table 3**.

The identified compounds from different fractions were then paired with antibacterial activity. Principal Component Analysis (PCA) divided all the compounds into two different groups: (I) with positive concentration coefficient; and (II) negative concentration coefficient. Positive coefficient indicates the increased extracted yields of specific compound with more concentrated ethanol solvents. However, compounds with negative concentration coefficient were more likely to be efficiently isolated with solvents of lesser ethanol concentrations. Bioactive compounds recorded varied antibacterial coefficient against *S. aureus* according to PCA. Compound 9 and compound 12 exhibited the highest antimicrobial coefficient (0.78 and 0.75, respectively), but compound 12 had the positive value (0.48) of concentration coefficient. Whereas, compound 9 had a negative concentration coefficient (-0.49) revealing the opposite extraction behavior than compound 12. Compound 10 was the third most active compound against *S. aureus*. Keeping in view only the compounds with positive concentration coefficient, compound 7 was the second most active compound against *S. aureus*, while compound 2 was the least active in this group. Among the compounds with negative concentration coefficient, compound 8 showed the least antibacterial activity against *S. aureus*. There were 13 compounds which stood neutral against the bacterial species, however, a total of 5 compounds were possessing negative coefficient values for controlling bacterial growth (**Figure 2A**).

Compounds 8, 9, and 10 were the most active compounds against *E. coli* among the group with negative concentration coefficient. Whereas, compound 7 recorded a comparable antibacterial coefficient to the compound 9, but with positive concentration coefficient value. Only one compound (29) didn’t affect the bacterial growth, while a total of 10 compounds exhibited negative antibacterial coefficient. Among those 10 compounds 3 had positive concentration coefficient and 7 exhibited negative concentration coefficient. The maximum negative antibacterial coefficient was shown by the compound 6 closely followed by compound 2. Collectively, the compounds showing substantial antibacterial coefficient were 7, 8, 9, and 10 (**Figure 2B**).

Compound 10 was the leading compound in controlling bacterial species *S. epidermidis* closely followed by compound 9. Compound 8 was the third compound among the group of compounds with negative concentration coefficient; however, it occupied overall fourth position in terms of antibacterial coefficient closely following compound 7. Keeping in view the low antibacterial activity, compound 6 showed the least coefficient, which was close to the values of compounds 1, 2, and 16. However, compound 16 was categorized among the compounds with negative concentration coefficient (**Figure 2C**).

Compound 10 occupied the highest antibacterial coefficient value with reference to *B. cereus* controlling activity, among all the detected compounds. Moreover, compound 9 and compound 8 possessed the second and third highest antibacterial activity in the group with negative concentration coefficient. However, compound 7 recorded the overall second position in comparison to all other compounds; but, its position among group of compounds with positive concentration coefficient was first. Compound 1, 2, and 6 had the lowest coefficient values for controlling bacterial species *B. cereus* in the positive concentration coefficient group. Whereas, among the compounds with negative concentration coefficients, compound 16 and 19 occupied the eminent position due to their lowest antibacterial activity (**Figure 2D**).

No compound detected in UPLC/MS could affect the growth of bacterial species *B. subtilis* and *P. aeruginosa*, hence producing zero coefficient values for their antibacterial activities. The only distribution of compounds could be seen on the axis of concentration coefficient revealing the differential extraction behavior of compounds against ethanol concentration (**Figures 2E,F**).

**FIGURE 3:**
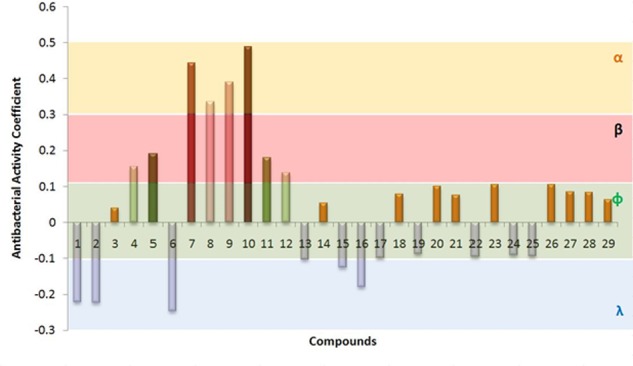
Cumulative antimicrobial activity of compounds determined from scores of Principal Component Analysis (PCA) against 6 bacterial species, i.e., *Staphylococcus aureus, Escherichia coli, Staphylococcus epidermidis, Bacillus cereus, Bacillus subtilis*, and *Pseudomonas aeruginosa*. All the compounds are single origin, seed coat of *Trachycarpus fortunei.* Compounds were classified into four groups (α, β, ф, and λ) by their antibacterial coefficient values determined by PCA. Compounds have been mentioned on X-axis are numbered from 1 to 29. Details of compounds are presented in **Table 3**.

Based on the above analysis, all the compounds were grouped into four different categories, i.e., α, α, ф, λ (**Figure 3**). The highest anti-bacterial activity was shown by α group. This group contains four different compounds having activity coefficient ≤ 0.03. Based on PCA analysis, compound 10 possess maximum anti-bacterial activity followed by compounds 7, 9, and 8. Four of these compounds (among three of them were not previously reported for antibacterial activity) were selected to test anti-bacterial activity by direct exposure to six bacterial strains. The β group showed closely lower anti-bacterial activity than α, and it also contained four member compounds 4, 5, 11, and 12. These compounds can be potentially used as anti-bacterial agents. However, they were screened and not tested for confirmation of their anti-bacterial potential in the current study. The 16 members of ф group accounted for slightly positive or negative behavior against bacterial species. Meanwhile, five compounds fall under λ category and they attributed negative anti-bacterial coefficient value suggesting their neutral to supporting behavior for bacterial pathogens according to the results of PCA.

**FIGURE 4:**
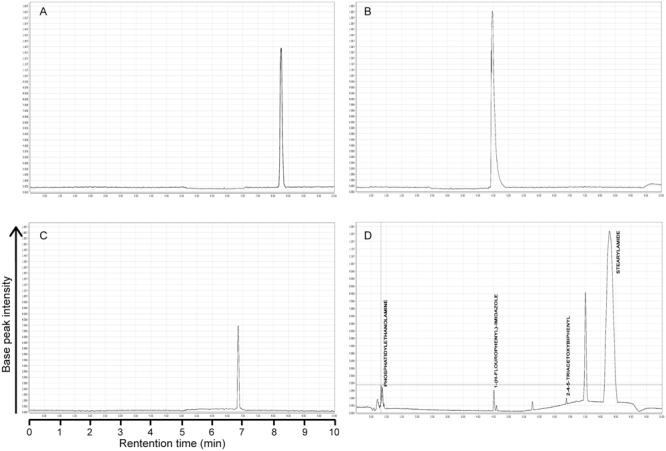
UPLC chromatograms of pure standard compounds and 20% ethanolic fraction of seed coat materials of *T. fortunei*. **(A)** Chromatogram of compound # 7 (Stearamide), **(B)** Chromatogram of compound # 9 (1-(4-Fluorophenyl)-2-(methylthio)-1H-imidazole-5-carboxylic acid), **(C)** Chromatogram of compound # 10 (2,4,5-triacetoxybiphenyl), **(D)** Chromatogram of 20% ethanolic fraction of seed coat materials of *T. fortunei*.

**FIGURE 5:**
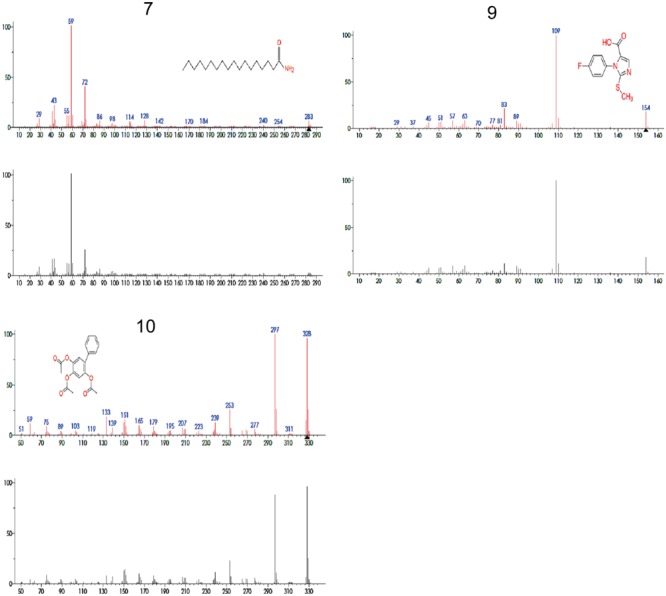
Mass spectra of compounds 7, 9, 10 that were identified by UPLC/MS analysis from 20% ethanol fraction of seed coat material of *T. fortunei*. 7 = Stearamide, 9 = 1-(4-Fluorophenyl)-2-(methylthio)-1H-imidazole-5-carboxylic acid, 10 = 2,4,5-triacetoxybiphenyl.

Principal component analysis generated antimicrobial profiles of UPLC/MS identified four different compounds, i.e., 7, 8, 9, and 10 having significant antimicrobial activity against most of the important bacterial pathogens. Among these four, three compounds are not previously reported for their antibacterial activity, while compound 8 anti-microbial activities has been previously reported (Murthy et al., 2011). Therefore, we used rest of the three compounds 7, 9, and 10 that were not previously reported for further study (**Figure 5**). To ensure the exact identification of the compounds UPLC/MS run of their individual standards were carried out. The peaks of the UPLC/MS run of standard chemicals were the same as found in UPLC/MS chromatogram of 20% ethanol fraction (**Figures 4, 5**).

### Antibacterial Compounds from 20% Ethanol Fraction of Seed Coat Extract of *T. fortunei*

Further, we evaluated antibacterial activity of pure bio-active compounds of fraction 20% ethanol. Compounds selection was made based on their peak area by UPLC/MS and activity of different fractions against bacterial strains. PCA was used for selection of compounds with significant antibacterial potential.

**FIGURE 6:**
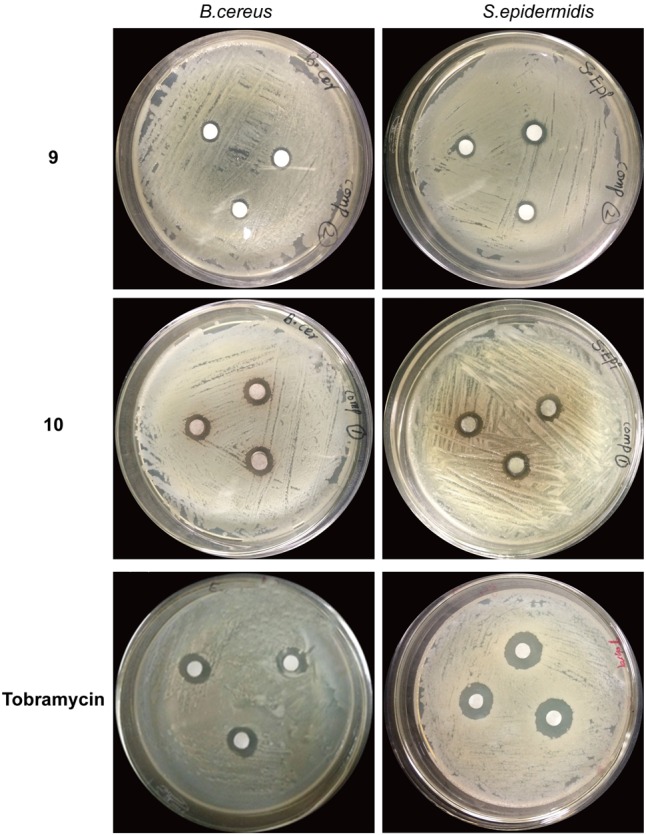
Antibacterial activity of compound 9 and 10 against Bacillus cereus and *Staphylococcus epidermidis*. Each chemical was used in quantity of 5 mg. Wells were inoculated with bacteria and incubated for 22 h at 37°C. Tobramycin was used as the positive control. 9 = 1-(4-Fluorophenyl)-2-(methylthio)-1H-imidazole-5-carboxylic acid, 10 = 2,4,5-triacetoxybiphenyl.

Compound 10, isolated from fraction 20% ethanol showed maximum antibacterial activity against all the bacterial strains followed by compound 9, whereas compound 7 was unable to inhibit the growth of any bacterial strain (**Table 4**). Compound 10 showed varied antimicrobial activity against all test bacterial strains. The maximum inhibitory zone (14 mm) was seen against *S. epidermidis.* This was followed by *B. cereus* by showing an inhibitory zone of 13 mm as shown in **Figure 6**. Likewise, compound 9 also showed maximum inhibitory potential against *S. epidermidis* and provided inhibition zone of 13 mm. In the same way, inhibition zone of 11 mm was observed when this compound was used against *B. cereus* as shown in **Table 4**. Altogether, the inhibitory activity of pure compounds was found less as compared to the parental 20% ethanol fraction.

**Table 4:** Antibacterial activity of identified compounds present in 20% ethanol fraction of *T. fortunei*.

Zone of inhibition (mm diameter)								
**Compound**	***S. aureus***		***E. coli***		***S. epidermidis***		***B. cereus***	
	**Dose (mg/ml)**		**Dose (mg/ml)**		**Dose (mg/ml)**		**Dose (mg/ml)**	
	**2.5**	**5**	**2.5**	**5**	**2.5**	**5**	**2.5**	**5**
7	N. A	N. A	N. A	N. A	N. A	N. A	N. A	N. A
9	8.67 ± 0.57	10 ± 1.73	8.5 ± 0.5	9.33 ± 0.57	11 ± 1	13 ± 1	9 ± 0	11 ± 1
10	9.33 ± 0.57	11.67 ± 0.57	8.67 ± 0.57	9.67 ± 1.52	12.17 ± 0.28	14 ± 0.5	10.67 ± 0.57	13.33 ± 0.57
P. control	17		17.5		19		17	
N. control	N. A		N. A		N. A		N. A	

*N. A, no activity; P. control, positive control (Streptomycin and tobramycin). N. control, Negative control (DMSO).*

The compound 10 exhibited a minimum MIC value (39.06 μg/mL) against *S. epidermidis* followed by *B. cereus* and *S. aureus* with MIC value of 156.25 μg/mL for both bacterial strains. In case of compound 9 the MIC value was a little higher for most of the bacterial strains, i.e., (312.5 μg/mL) as shown in **Table 5**.

**Table 5:** Minimum inhibitory concentration (MIC, μg/mL) of compounds present in 20% ethanolic fractions of seed coat material of *T. fortunei* against different bacteria.

Compounds	*S. aureus*	*E. coli*	*S. epidermidis*	*B. cereus*
9	312.5	312.5	312.5	625
10	156.25	312.5	39.06	156.25

*9, 1-(4-Fluorophenyl)-2-(methylthio)-1H-imidazole-5-carboxylic acid; 10, 2,4,5-triacetoxybiphenyl.*

MMC values of the pure compounds of active fraction 20% ethanol against reference bacterial strains were shown in **Table 6**. Although results of MIC and MMC showed variations among the tested bacterial strains but both the values of MIC and MMC were equivalent with each other in most of the cases. Compound 10 showed the least value of MMC (78.25 μg/mL) against *S. epidermidis* followed by *B. cereus* with MMC value of 625 μg/mL. Whereas, compound 9 showed its antibacterial potential against all the tested microbial species at a constant MMC value of 312.5 μg/mL.

**Table 6:** Minimum microbicidal concentration (MMC, μg/mL) of compounds present in 20% ethanolic fraction of seed coat materials of *T. fortunei* against different bacteria.

Compounds	*S. aureus*	*E. coli*	*S. epidermidis*	*B. cereus*
9	1250	1250	1250	1250
10	1250	1250	78.25	625

*9, 1-(4-Fluorophenyl)-2-(methylthio)-1H-imidazole-5-carboxylic acid; 10, 2,4,5-triacetoxybiphenyl.*

## Discussion

Indiscriminate use of commercial antimicrobial drugs and poor patient’s compliance have led to the development of bacterial resistance to antibiotics. Current scenario has forced scientists to search for new antimicrobial substances with novel mode of actions. The investigation of the efficacy of plant based drugs has gain great attention because of fewer side effects, low cost and ace of availability. In this study, we have screened and identified antibacterial agents from seed coat material of *T. fortunei.* Mostly identified antimicrobial components from plants are aromatic or saturated organic compounds, requiring initial ethanol or methanol extraction (Gilbert and Alves, 2003). Therefore ethanol was used for the preparation of crude extracts and for subsequent fractions preparations. After preliminary assessment of antibacterial activity, the crude ethanol extracts were subjected to bio-guided fractionation by sequential partition with different concentration of aqueous ethanol solvent system and yielding four fractions (0, 20, 40, and 60% ethanol). These four different ethanolic fractions of seed coat extracts were tested against selected human pathogenic bacterial strains belonging from different genera. All the fractions showed varying levels of antibacterial activity against different bacterial strains. However, antibacterial assay of 20% ethanol fraction (5 mg) showed maximum effectiveness against most of the tested bacterial strains including *E. coli, S. aureus, S. epidermidis, B. cereus.* Previously, there have been some reports of the antibacterial and antifungal activities of different parts of *T. fortunei* (Essig and Dong, 1987). However, this study is the first report of antimicrobial activity of seed coat materials of *T. fortunei*.

Furthermore, MIC assay for quantitative study was performed to find out antibacterial activity of different ethanol and aqueous fractions of seed coat materials of *T. fortunei*. Twenty percent ethanol fraction showed the lowest MIC value (156.25 μg/ml) among all the tested fractions against *E. coli* and *S. aureus* (**Table 1**). Phytochemicals are usually grouped as antimicrobials if MIC values range between 100 and 1000 mg/mL (Simoes et al., 2009). In the same way, activity of crude extract will be considered significant if MIC values are <100 μg/mL (Kuete, 2010). In lieu of that, our findings can considered as noteworthy. Furthermore, extracts and natural products possessing MIC values < 8 mg/ml are considered significant resources for the discovery of new medicines against different infectious diseases (Fabry et al., 1998; Gibbons, 2004; Rios and Recio, 2005). Therefore, the recorded antibacterial activity of 20% ethanolic fraction can be considered as significant. Secondly, all fractions showed anti-bacterial activity when used in less quantity as compared to the quantity of antimicrobial agent used as positive control against all the tested microbial strains.

In order to screen the bio-active compounds from a mixture, the classic way involves compounds separation followed by activity analysis at each subsequent step. However, this may take a long time. In this study we adopted a different strategy to screen anti-bacterial chemicals. We performed UP-LC/MS analysis and correlated identified biochemical richness with anti-bacterial activity of parent fractions by performing principal component analysis. This approach has been successfully used in some previous studies to find active biochemicals. Keller et al. (2013) used similar approach to find putative antibacterial biochemicals from fermented *Camellia sinensis* tea. Candidate compounds were identified by analysis between two tea samples after performing their UPLC/MS analysis for identification and quantification of chemical compounds. Afterward, abundance of identified compounds was compared with antibacterial activity of tea samples to find putative active bio-chemicals from a large group of dataset (Keller et al., 2013). Here we adopted similar strategy to find out putative anti-bacterial compounds from different parent fractions of seed coat extracts of *T. fortunei*.

UPLC/MS analysis identified four compounds in fraction with maximum antibacterial activity (20% ethanol) that were named in numerical codes, i.e., compound 7 (Stearamide), 8 (imidazole), 9 (1-(4-Fluorophenyl)-2-(methylthio)-1H-imidazole-5-carboxylic acid) and 10 (2,4,5 triacetoxybiphenyl). Compound 8 has been previously reported for its anti-bacterial activity (Murthy et al., 2011) and was skipped from present investigation. The rest three compounds (7, 9, and 10) were obtained commercially and tested for their antibacterial activity. Two compound “9 and 10” showed anti-bacterial activity against most of the test bacterial species. However, Compound 8 was unable to inhibit bacterial growth at any test concentration (**Table 4**).

A considerable number of pyrazolos including imidazole are known to display microbicidal activities. These compounds have amino acid moieties and possess a wide range of biological and pharmacological activities (Song, 2008; Jain et al., 2010;Karakurt et al., 2010). Two compounds, i.e., 8 and 10 contain imidazole groups. Imidazole derivates target cholesterol backbone resulting in a disruption of the outer membrane and subsequent cell death (Ding et al., 2004). Compound 9 belongs to biphenyls. These compounds are bacterial adhesive component “FimH” antagonists that is required to colonize and invade the host cells during infection process (Waksman and Hultgren, 2009). FimH mediates the colonization and invasion of the bladder epithelium during Escherichia coli urinary tract infections (Rosen et al., 2008).

The results of MIC assay showed that these compounds significantly suppressed the growth of test bacterial strains (**Table 5**). The lowest MIC value (39.06 μg/mL) was seen for compound 10 against bacterial strain “*S. epidermidis.”* The activity noted can be considered as noteworthy, in view of the cutoff value “100 μg/mL” that is considered necessary for MIC values of plants extracts having significant mode of action against different microbes (Kuete, 2010). The activity of compound 10 can be considered as moderate (Kuete, 2010) which represents 25% of the checked microorganisms in this research. Ethanolic fractions showed increased antibacterial activity as compared to pure compounds, which might be due to inseparable compounds, synergetic effect, labile constituents, multiple weakly active constituents or poor separation of the bioactive compound (Gilbert and Alves, 2003; Rasoanaivo et al., 2011).

## Conclusion

The antibacterial activity of different parts of *T. fortunei* was previously reported, yet antibacterial activity of seed coat material had never been assessed before. Thus, this is the first report of screening and identification of antibacterial compounds seed coat material of this plant species. These compounds were effective against some human pathogenic bacterial strains who are involved in many serious human diseases like pneumonia and urinary tract infections etc. Furthermore, these compounds appear to be different from already known constituents of this plants and hence its bactericidal activity can be further explored.

## Author Contributions

Conceived and designed the experiments: XH and SA. Performed the experiments: SA, HL, AA, WA, EA, QZ, FR, WO, and XL. Analyzed the data: SA, AA, WA, QC, and XH. Performed sample preparation and experiments: SA, HL, AA, WA, EA, QZ, SD, and XL. Wrote the manuscript: SA, AA, SH, and XH.

## Conflict of Interest Statement

The authors declare that the research was conducted in the absence of any commercial or financial relationships that could be construed as a potential conflict of interest.
